# An engineered pathway for the biosynthesis of renewable propane

**DOI:** 10.1038/ncomms5731

**Published:** 2014-09-02

**Authors:** Pauli Kallio, András Pásztor, Kati Thiel, M. Kalim Akhtar, Patrik R. Jones

**Affiliations:** 1 Department of Biochemistry, University of Turku, Tykistökatu 6B 4krs, 20520 Turku, Finland; 2 Department of Life Sciences, Imperial College London, Sir Alexander Fleming Building, London SW7 2AZ, UK; 3 These authors contributed equally to the work; 4 Present address: Department of Biochemical Engineering, University College London, Torrington Place, London WC1E 7JE, UK

## Abstract

The deployment of next-generation renewable biofuels can be enhanced by improving their compatibility with the current infrastructure for transportation, storage and utilization. Propane, the bulk component of liquid petroleum gas, is an appealing target as it already has a global market. In addition, it is a gas under standard conditions, but can easily be liquefied. This allows the fuel to immediately separate from the biocatalytic process after synthesis, yet does not preclude energy-dense storage as a liquid. Here we report, for the first time, a synthetic metabolic pathway for producing renewable propane. The pathway is based on a thioesterase specific for butyryl-acyl carrier protein (ACP), which allows native fatty acid biosynthesis of the *Escherichia coli* host to be redirected towards a synthetic alkane pathway. Propane biosynthesis is markedly stimulated by the introduction of an electron-donating module, optimizing the balance of O_2_ supply and removal of native aldehyde reductases.

## Abstract (web summary)

Propane is the main component of liquid petroleum gas and has a wide variety of commercial applications. Here, the authors engineer a synthetic metabolic pathway in *E. coli*, and demonstrate for the first time the renewable production of propane.

## Graphical abstract

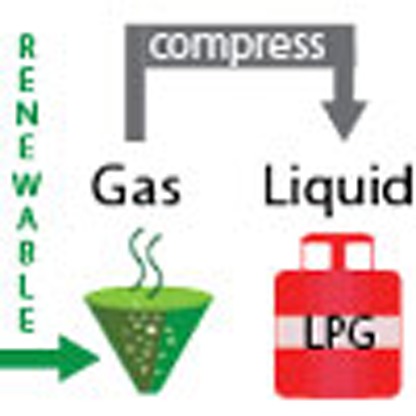

The global market for fuel (~85% of total energy use) is enormous, yet the unit value is very low. This places a harsh constraint on the development of an economically sustainable technology that can complement and subsequently replace petroleum-derived fuels. Renewable biofuels currently contribute only ~0.5% of global fuel supply^1^, yet, the limit for blending ethanol in petrol-based combustion engines has in regions like the United States already been reached^2^. A major, and often unanswered, question is ‘which biofuel to produce?’ The ‘best’ choice is not obvious considering all constraints for biological, chemical and motor engineering, each one setting demands for future sustainable processes, from production to utilization.

A characteristic feature of microbial hydrocarbon fuel platforms developed so far is that the end-products are in their liquid states under atmospheric conditions (Supplementary Table 1). This is problematic for two main reasons. First, direct physical interaction with the fuel end-product can be detrimental to the growth or metabolism of the host^3^. This has prompted further investigations into enhancing host tolerance towards toxic end-products^4^. Second, separation and purification of the fuel from the liquid media or the biological host (if confined to the intracellular compartment) necessitates the use of complex and/or costly techniques^5^. To enhance the sustainability and industrial adoption of renewable fuels in the future, we would suggest the following criteria to be important to consider: (i) reduced toxicity of the end-product to the biotechnological host, (ii) immediate separation of the fuel end-product from the manufacturing process, (iii) facile storage of the fuel in an energy-dense liquid state, (iv) compatibility with existing infrastructure for utilization and (v) compatibility with future photobiological processes.

We propose that propane (C_3_H_8_) would satisfy well the above criteria. Propane is a bulk constituent of liquefied petroleum gas with an existing global market for a wide number of stationary and mobile applications (heaters, gas burners, refrigeration systems and spark-ignition engines). An attractive feature of propane is its facile phase change between gaseous and liquid states at ambient conditions. This allows easy separation from the liquid-based biotechnological process as a gas, yet does not preclude post-production storage and transport in a high-energy-density liquid state. The latter attribute is a central issue limiting the utility of H_2_, as it requires substantial energy to be liquefied (30-fold more compared with propane, Supplementary Table 1). Immediate separation of the end-product during the manufacturing process offers also other potential advantages including lower host toxicity, enhanced metabolic pathway thermodynamics and use of the biomass as a continuously operating catalytic system. If such a process could be coupled to high-efficiency conversion of solar to chemical energy, opportunities for renewable fuel production could be far-reaching.

As far as we are aware, however, no metabolic pathway exists for the renewable biosynthesis of propane. The discovery of the aldehyde deformylating oxygenase (ADO; formerly aldehyde decarbonylase) enzyme family in cyanobacteria^6^ has opened up the door for engineering metabolic pathways for alkane biosynthesis. In the presence of an electron donor, ADO catalyses the O_2_-dependent conversion^7^ of aldehyde into alkane and formate^8^ with native hydrocarbon chain-lengths in the range of C15–C17. Several variations of alkane pathways have already been engineered in *E. coli*^6^,^9^,^10^,^11^,^12^ using either ADO or an enzyme called CER1 from higher plants^12^. These microbial platforms, which mainly differ in the chain length of their aldehyde precursors, have led to the production of medium chain-length alkanes (C15–C17 (ref. 6), C13–C17 (ref. 9), C11–C17 (ref. 10), C13–C17 (ref. 11), C9–C14 (ref. 12)) in *E. coli*.

In this study, we demonstrate a microbial platform for the production of propane (C_3_H_8_) by engineering a synthetic metabolic pathway that is dependent on fatty acid biosynthesis (Fig. 1). The pathway is designed to operate in the presence of oxygen in order to allow future implementation within an oxygenic, photosynthetic host such as cyanobacteria. Through systematic assembly and evaluation of this platform, we reveal biochemical and environmental factors, which are critical for optimal *in vivo* propane production with likely consequences for the biosynthesis of any alkane.

## Results

### Engineering a synthetic pathway for propane biosynthesis

**Figure 1:**
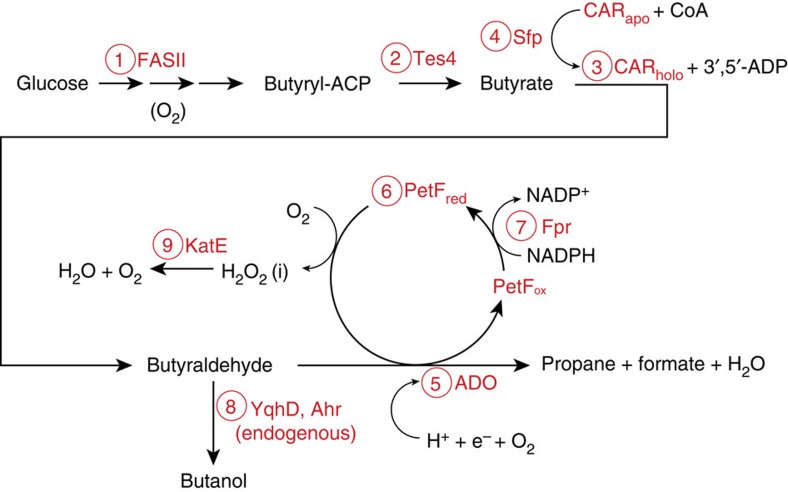
Schematic (non-stoichiometric) representation of the constructed heterologous propane pathway in *E. coli* BL21 (DE3). The associated enzyme components are shown in red: (1) butyryl-ACP is generated via the native fatty acid biosynthesis (FASII) pathway, followed by (2) the release of butyrate by thioesterase (Tes4; *Bacteroides fragilis)*. (3) Butyrate is converted into butyraldehyde by carboxylic acid reductase (CAR; *Mycobacterium marinum*) with the aid of a (4) maturase phosphopantetheinyl transferase (Sfp; *Bacillus subtilis*). (5) The butyraldehyde intermediate is finally converted to propane by aldehyde deformylating oxygenase (ADO; *Prochlorococcus marinus*), whereas (6) ferredoxin (PetF; *Synechocystis sp.* PCC6803) provides electrons for the reaction. (7) Ferredoxin in this pathway is reduced by overexpressed NADPH:ferredoxin/flavodoxin-oxidoreductase (Fpr; *E. coli*). (8) Endogenous aldehyde reductases (YqhD, Ahr) compete with ADO for the butyraldehyde substrate and reroute the desired pathway towards butanol. (9) Overexpressed catalase (KatE; *E. coli*) converts inhibitory hydrogen peroxide to oxygen and water.

The final precursor for propane biosynthesis is butyraldehyde. Several *Clostridium* species naturally harbour a CoA-dependent pathway for 1-butanol synthesis that also proceeds via a butyraldehyde intermediate^13^. The final step in 1-butanol biosynthesis is catalysed by AdhE2, a bi-functional acyl-CoA/aldehyde reductase^14^, using butyryl-CoA as the immediate precursor. As AdhE2 directly converts butyryl-CoA to 1-butanol, without releasing the butyraldehyde intermediate needed for the ADO reaction, alkane biosynthesis would not be possible with an AdhE2-dependent pathway. In addition, AdhE2 is reported to be sensitive to O_2_ (ref. 14) an essential co-substrate for ADO^7^. Although an O_2_-tolerant butyraldehyde dehydrogenase (PduP) was recently shown to generate free butyraldehyde in cyanobacteria^15^, we instead exploited an ATP-dependent carboxylic acid reductase (CAR; Fig. 1; step 3) that catalyses the conversion of a broad range of fatty acids to their corresponding aldehydes, including butyrate to butyraldehyde^10^.

**Figure 2:**
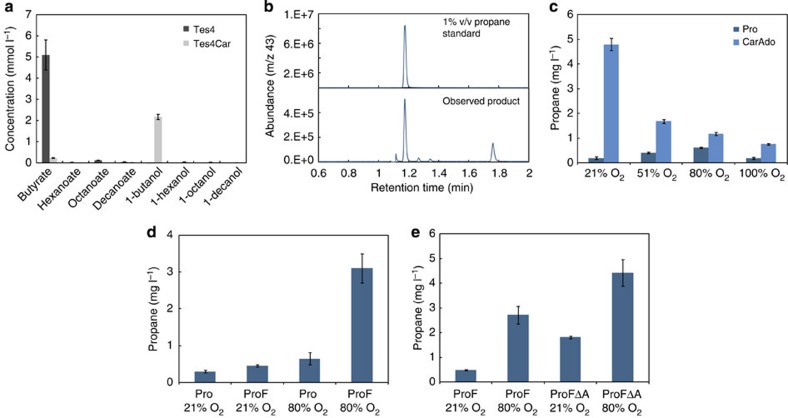
Propane and metabolite production under various environmental conditions using different strains. (**a**) Tes4 and Tes4Car strains were compared for fatty acid and alcohol production in shake flask fermentation under aerobic conditions; metabolites were analysed from culture supernatant after 24 h cultivation. (**b**) GC–MS chromatogram of a typical sample injected from the headspace of the propane-producing strain ProFΔA and a 1% (v/v) propane standard. (**c**) Supplementing the reaction culture headspace with oxygen to the final concentration of 51, 80 and 100% (v/v) using Pro strain (generating propane from glucose) and CarAdo strain (fed with butyrate). (**d**) Co-expression of Fpr and PetF with and without O_2_ supplementation using strains Pro and ProF. (**e**) The effect of aldehyde reductase knock-outs with and without O_2_ supplementation with strains ProFΔA versus ProF. Error bars, mean±s.d. (*n*=4).

In order to supply butyrate for butyraldehyde synthesis, we sought to redirect native fatty acid biosynthesis (FASII; Fig. 1; step 1) of the host as demonstrated earlier for medium-chain alcohols and alkanes^10^. To achieve this, a thioesterase with high specificity for C4 butyryl-ACP was required. Heterologous expression of a bacterial acyl-ACP thioesterase from *Bacteroides fragilis* (Tes4) in *E. coli* K27 was recently reported to result in the accumulation of low quantities of butyrate, although >85% of the identified products were longer chain-length acids such as 8:0, 12:1 and 14:1 (ref. 16). Surprisingly, in our hands the expression of Tes4 (Fig. 1; step 2) in *E.coli* BL21(DE3) (Supplementary Table 2; strains Tes4 and Tes4Car) resulted in accumulation of over 5 mM butyrate as the main product, whereas only low amount of octanoate and minor quantities of >C8 chain-length acids were detected (Fig. 2a, Supplementary Fig. 1). There are two main differences between our experiments and those of Jing *et al.*^16^, which may explain the contrasting results. First, they used an *E. coli* K strain, which differs markedly^17^ from the B strain we used in our study. Second, FadD, an acyl CoA synthetase that catalyses the first step in the fatty acid β-oxidation pathway, and which therefore may compete for the fatty acid substrate of CAR, is missing in K27. Although the deletion of *fadD* was earlier found to be beneficial for free fatty acid production in a BL21(DE3) derivative strain using the C12–C16-specific *E. coli* thioesterase TesA^18^, recent studies using the same thioesterase and strain background suggested the opposite^19^. Therefore, the marked difference in the product profile between our work and the study of Jing *et al.*^16^ remains unknown and in need of further studies.

Nevertheless, the availability of a C4-specific thioesterase allowed the assembly of a synthetic pathway for propane biosynthesis (Fig. 1) in BL21(DE3) by co-expression of Tes4 with CAR (*Mycobacterium marinum*)^10^, the CAR maturation factor phosphopantetheinyl transferase (Sfp*; Bacillus subtilis*)^20^, ADO (*Prochlorococcus marinus*)^6^ and ferredoxin (PetF; *Synechocystis sp.* PCC6803; Supplementary Table 2; strain Pro). The 2Fe-2S ferredoxin (PetF) (Fig. 1; Step 6) from cyanobacteria was introduced in order to enhance electron-supply, as the availability of ferredoxin or ferredoxin-like electron acceptor/donors was previously observed to be inadequate for a synthetic H_2_-pathway in *E. coli* BL21(DE3)^21^. A gas-tight cultivation method was used in order to allow propane to accumulate in the reaction vial headspace. Careful optimization of the analytical setup enabled reproducible gas chromatograph–mass spectrometer (GC–MS)-based quantification of even subtle changes in product formation. Mass fragment product analysis of cultures fed with ^13^C-labelled glucose confirmed that the synthesized propane (Fig. 2b) was derived from glucose supplemented to the medium (Supplementary Fig. 2).

### Propane synthesis is dependent upon oxygen availability

Although the FASII-dependent propane pathway in the Pro strain was functional, continuous production could not be maintained and a plateau was reached within an hour after the culture vials were sealed (Supplementary Fig. 3). A similar trend was also noted for the pathway intermediate, butyrate (Supplementary Fig. 4A), in addition to the unwanted side-product, 1-butanol (Supplementary Fig. 4B). In order to understand and address the limiting factors affecting propane biosynthesis, a systematic evaluation of the effect of precursor supply, O_2_ availability, H_2_O_2_-metabolism, electron-supply and competing native pathways was carried out using a combination of different strains (Supplementary Tables 2 and 3) and conditions (Supplementary Table 4).

Propane synthesis in the closed-vial systems was enhanced by the presence of additional O_2_ (Fig. 2c, Supplementary Fig. 5A), increased relative head-space volume (Supplementary Fig. 6), or periodic regeneration of the head-space with air (Supplementary Fig. 7). This response to O_2_ was unlikely due to changes in cell density as additional supply of oxygen had a negligible effect on growth during the reaction (Supplementary Fig. 5B), and increasing the cell density in the sealed vials had very little impact on propane production (Supplementary Fig. 8). When butyrate was supplied directly to the culture medium (Supplementary Table 2; strain CarAdo), the plateau in propane accumulation was delayed (Supplementary Fig. 9) and yields were higher (Fig. 2c). Together these findings suggested that (i) the limitation observed with glucose-dependent propane biosynthesis in the closed system was caused by insufficient availability of butyrate and not the downstream biosynthetic steps, and (ii) the amount of FASII-derived butyrate was limited by the availability of oxygen. This hypothesis was further corroborated experimentally by demonstrating that the pool of free butyrate (Supplementary Table 2; strain Tes4) increased in relation to the amount of supplied O_2_ (Supplementary Fig. 10). In parallel, lack of O_2_ may also directly limit the ADO-catalysed conversion of butyraldehyde to propane as was previously demonstrated *in vitro*^7^. This may explain why complete depletion of O_2_ from the culture by flushing with N_2_ severely compromised propane biosynthesis even in the presence of butyrate (Supplementary Fig. 11).

Even though oxygen is a prerequisite for alkane biosynthesis, an excess supply of pure O_2_ was found to have a negative impact on production (Fig. 2c). As ferredoxins have been shown to generate superoxide radicals^22^ and ADO can be inactivated in the presence of H_2_O_2_ (ref. 23), we hypothesized that the increase in O_2_ content, and/or the effect of O_2_ on electron supply, interferes with ADO activity through an increase in H_2_O_2_. One possible strategy for resolving this issue is to over-express a catalase, which breaks down H_2_O_2_, as was shown previously^23^. Despite a comprehensive evaluation, however, we did not observe any clear positive effect on alkane accumulation by over-expression of the catalase KatE (Fig. 1; Step 9) from *E. coli* (Supplementary Fig. 12).

### Increased electron supply enhances propane synthesis

For the conversion of aldehydes to alkanes, ADO forms a peroxo-intermediate as part of its reaction mechanism^24^,^25^. This requires an input of four electrons for every alkane that is formed; this electron supply chain has been previously reconstituted (non-chemically) *in vitro* by the use of non-native surrogate ferredoxins and/or ferredoxin reductases^6^,^7^,^23^,^26^ and recently, by a combination of native enzymes from *Synechococcus elongatus* PCC7942 (ref. 27). The previously reported *in vivo E. coli* systems, however, have relied exclusively upon the endogenous capacity of the host for ADO reduction^6^,^9^,^10^,^11^. In order to provide sufficient supply of electrons to ADO in our system, 2Fe-2S ferredoxin PetF from *Synechocystis sp*. PCC 6803 was over-expressed as part of the pathway. In parallel, to ensure that insufficient reduction of ferredoxin was not limiting pathway flux as was previously observed with a synthetic H_2_-pathway^21^, a ferredoxin-oxidoreductase was further introduced into the system. Even though native cyanobacterial redox partners have been shown to confer slightly higher ADO activity *in vitro*^27^, the *E. coli* counterpart was selected for this study for maximal compatibility with the overexpression host. In *E. coli,* there are two annotated flavodoxin/ferredoxin-oxidoreductases: (i) the pyruvate/ferredoxin-oxidoreductase YdbK and (ii) NADPH/ferredoxin/flavodoxin-oxidoreductase Fpr. The extreme O_2_-sensitivity of YdbK^28^ ruled out its involvement in the aerobic formation of propane leaving Fpr as the most likely endogenous candidate. The overexpression of Fpr (Fig. 1; step 7) resulted in only a small increase in propane under standard conditions (Fig. 2d; 21% (v/v)). In contrast, when the O_2_ concentration was increased to 80% (v/v), there was a five- to eightfold increase in propane accumulation, enhancing the titre to over 3 mg l^−1^ of propane (Fig. 2d, Supplementary Figs 13–15).

### Competition with native host metabolism

**Figure 3:**
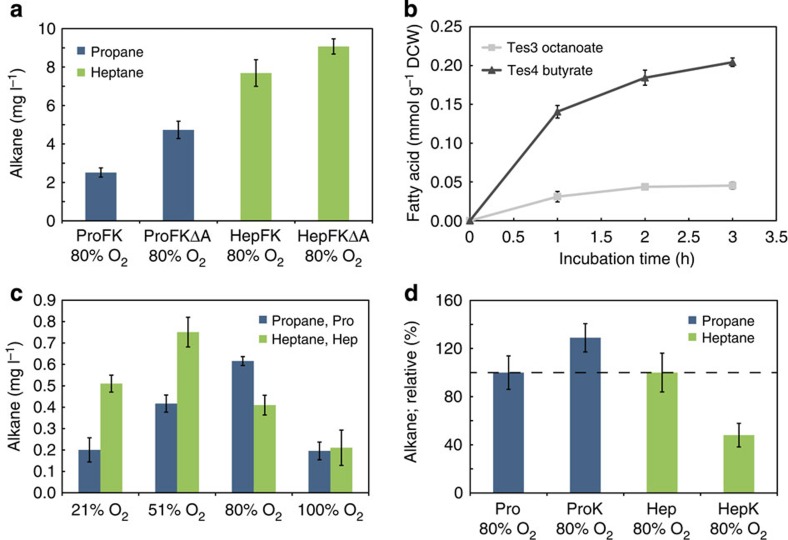
Comparison of the pathways for propane and heptane production. (**a**) The effect of aldehyde reductase knock-outs in ΔyqhD Δahr strains ProFKΔA and HepFKΔA versus the corresponding strains ProFK and HepFK, respectively, with supplemented oxygen. (**b**) Octanoate and butyrate production with strains Tes3 and Tes4, respectively, using conditions optimized for propane production (80% v/v oxygen). (**c**) Supplementing the reaction culture headspace with oxygen to the final concentration of 51, 80 and 100% (v/v) using strains Pro and Hep. (**d**) Overexpression of KatE in strains ProK and HepK versus Pro and Hep with supplemented oxygen; values are represented as relative % for comparison. Error bars, mean±s.d. (*n*=4).

*E. coli* has an efficient system for removing potentially harmful intracellular aldehydes by the action of aldehyde reductases and dehydrogenases. This native detoxification mechanism by the host is likely to compete with the ADO enzyme for the aldehyde substrate^29^. The accumulation of 1-butanol with the propane-generating strains Pro, and its parent strain Tes4Car (Fig. 2a, Supplementary Fig. 4B), suggested that this indeed was the case. In order to efficiently channel the butyraldehyde precursor towards propane, two aldehyde reductase genes *ahr* and *yqhD* (Fig. 1; Step 8) that had previously been shown to be most important for the conversion of isobutyraldehyde to isobutanol^29^ were deleted. The resulting strains (Supplementary Table 2; strains ProFΔA and ProFKΔA) showed enhanced propane synthesis at both atmospheric (21% (v/v)) and elevated oxygen concentrations (80% (v/v)), resulting in approximately three- and twofold improvement, respectively (Figs 2e and 3a). As expected, the increase in propane afforded by the aldehyde reductase deletions (Supplementary Fig. 16A) was followed by a concomitant decrease in the production of butanol (Supplementary Fig. 16B).

### Enzyme constraints for alkane biosynthesis

Although both the CAR and ADO enzymes can accept four-carbon substrates, earlier *in vitro* characterization suggest that they exhibit superior kinetics for longer chain substrates. For example, the *K*_M_ values for CAR are ~90 times lower for C8 versus C4 (ref. 10) whereas the *K*_app_ values for ADO are ~190 times higher for C8 versus C4 (ref. 30). In order to test whether poor enzyme kinetics was compromising the performance of the propane pathway *in vivo*, we constructed a C7 heptane pathway, by exchanging the thioesterase gene *tes4*, with *tes3* (*Anaerococcus tetradius*), that has been reported to generate primarily the C8 fatty acid, octanoate^16^. Induction of this modified pathway resulted in the accumulation of heptane (Supplementary Fig. 17A) from glucose (Supplementary Fig. 17B) within the headspace of the sealed cultivation vessels, although the majority of the product is expected to exist in the liquid state under the conditions employed. Using the optimal conditions established for propane synthesis, heptane titres were found to be almost twofold greater than propane (5 versus 9 mg l^−1^; Fig. 3a). However, the quantity of the heptane precursor (octanoate) released by the Tes3 strain was only about one-fourth of the amount of the propane precursor (butyrate) released by Tes4 (Fig. 3b), which confirmed that the kinetic properties of the CAR and ADO enzymes used in this study indeed were limiting propane productivity. The effect of supplemented oxygen on heptane production was similar as with propane, although the overall positive impact was less significant (<50% improvement) and inhibitory effects were observed at lower oxygen concentrations (Fig. 3c). Catalase over-expression did not improve heptane productivity and generally only had a slight negative effect (Fig. 3d, Supplementary Fig. 18). Co-expression of Fpr (Supplementary Fig. 19) and deletion of the aldehyde reductases (Supplementary Fig. 20), however, had a stimulating impact.

### Potential for large-scale production of propane

**Figure 4:**
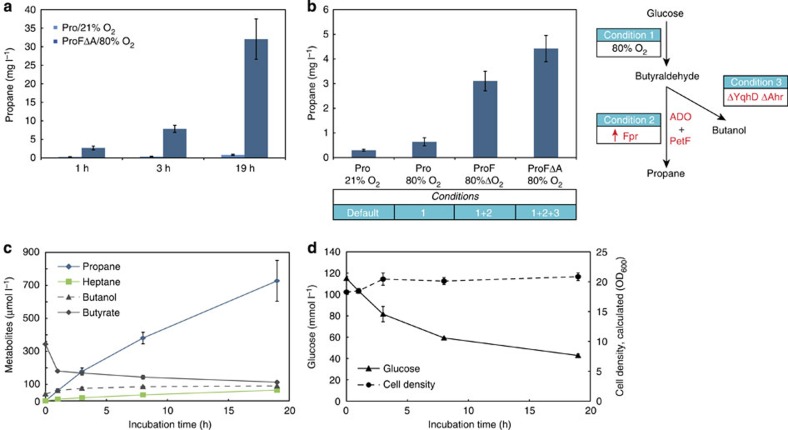
Optimization of propane production. (**a**) Scale-up of propane production. Cultures of strains Pro and ProFΔA were scaled up from 0.5 ml (2 ml vial) to 20 ml (in 160 ml serum bottles). 80% (v/v) O_2_ was supplemented to ProFΔA strain. (**b**) Step-wise improvement of propane production from glucose in *E. coli* BL21 (DE3) host (Bars from left to right): The default pathway at atmospheric oxygen concentrations (21% (v/v)); supplementing additional oxygen (80% v/v); overexpression of NADPH:ferredoxin/flavodoxin-oxidoreductase (Fpr from *E. coli*) with 80% (v/v) oxygen; deletion of the two native aldehyde reductase encoding genes, *yqhD* and *ahr*, together with fpr and 80% (v/v) oxygen. For detailed information on the metabolic pathway refer to Fig. 1. (**c**) Metabolite analysis of ProFΔA strain in up-scaled reaction conditions (160 ml serum bottles, 80% v/v O_2_) over a 19-h cultivation period. The moment of sealing, 4 h after induction, represents the time-point 0 h. The amounts of octanol and octanoate were below detection limit. (**d**) Glucose consumption and growth rate analysis of the same experiment. Error bars, mean±s.d. (*n*=4).

Throughout this study, cultivations were performed with small-scale cultures (0.5 ml) using gas-tight crimped GC vials (2 ml). Although this allowed high-throughput evaluation with a large number of replicates, it was obviously not ideal for evaluating the potential productivity. For example, termination of propane accumulation within the first 3 h of cultivation was repeatedly observed, which raised doubts about the longevity of the propane-generating system. To address this issue, while also assessing the system in larger batch cultures, the culture volume was scaled up 40-fold to 20 ml, whereas the volume of the cultivation vessel was increased 80-fold to 160 ml. Overall, this resulted in a greater headspace/liquid ratio (from 3:1 to 7:1) for which the gas-environment was expected to be less variable. Indeed, propane production continued up to 19 h, resulting in a total titre of 32 mg l^−1^ (Supplementary Table 2; strain ProFΔA and 80% (v/v) O_2_ supplementation; Fig. 4a). From the initial development of the propane-producing pathway, the overall titre was improved more than two orders of magnitude (from 0.3 to 32 mg l^−1^). The step-wise improvement in propane production from genetic modification to physical optimization is summarized in Fig. 4a,b. Figure 4c,d show the detailed production profile of the up-scaled system over a 19-h cultivation illustrating the parallel formation of the end-products (i) propane, (ii) heptane, the corresponding fatty acids (iii) butyrate and (iv) octanoate, the alcohols (v) butanol and (vi) octanol, respectively, in addition to (vii) glucose concentration and (viii) cell density. Interestingly, the accumulation of butanol stalled soon after the 8 h point, whereas propane accumulation continued and eventually became dominant over the other C4 products.

## Discussion

Although fracking has provided a boost in the supply of liquid and gaseous fossil fuels, there is still a continued need for genuinely sustainable energy technologies over the long-term^31^,^32^. A need to phase out current crop-based biofuels and move towards next-generation technologies that do not compete for food has also been raised^33^. In response to this and existing earlier concerns (greenhouse gas emissions, independent supply and so on), synthetic pathways for a large number of novel fuel molecules have recently been engineered^10^,^11^,^34^. Given the availability and low cost of fossil fuels, a major challenge for all novel renewable fuel production concepts is to achieve economically sustainable well-to-wheel systems. In order to maximize the chance for novel renewable technologies to be commercialized, we argue that it is important to identify an optimal process and molecular target with respect to the entire chain from CO_2_ to fuel and then eventually back to CO_2_ again following combustion, including separation, chemical processing, storage and distribution there in between. The best molecular choice for all these considerations is not obvious and with few exceptions^35^,^36^ a wide range of criteria have not been considered. For example, although butanol has higher energy density and lower water solubility compared with ethanol, it exhibits greater toxicity to biotechnological strains^37^. With the constraint of identifying a novel target that is well suited for the entire chain of production, distribution and utilization, propane was proposed for the following reasons: (i) alkanes are less toxic than their corresponding alcohols and acids^36^, (ii) the gaseous state of propane at common atmospheric conditions allows spontaneous separation during production, (iii) it can be stored in a high-density liquid state and (iv) it has excellent compatibility with the current fuel infrastructure for distribution and utilization (that is, liquefied petroleum gas).

We identified three main biochemical factors that limited the productivity of the synthetic propane pathway. First, the reduction of ADO was insufficient. This was demonstrated by reconstitution of an electron relay system to ADO, for the first time *in vivo*, by introducing ferredoxin and a NADPH/ferredoxin-oxidoreductase (Fpr), which together significantly enhanced propane synthesis. Although Fpr catalyses a thermodynamically unfavourable reaction (Δ_r_*G*′^0^=10.7 kJ mol^−1^ (ref. 38), it is the only known option for reduction of ferredoxin under aerobic conditions. For photobiological systems, however, we expect this to be less of an issue as ferredoxin is naturally reduced by photosystem I (ref. 39). Second, native aldehyde reductase activity was found to be a major competitor of alkane formation. This issue was addressed through removal of two host genes, *yqhD* and *ahr*, which are believed to account for the majority of the total aldehyde reductase activity^29^. As butanol still accumulated, the elimination of remaining aldehyde reductases is likely to stimulate alkane synthesis even further. Third, our results demonstrate that unfavourable kinetics of CAR and ADO limit propane biosynthesis *in vivo*. Thus, further improvement would be expected with enzyme variants or homologues with higher specificity towards C4 substrates^30^, particularly if implemented in combination with recent methods to enhance metabolic flux through the fatty acid biosynthesis pathway^40^.

The most important environmental factor for propane synthesis was found to be O_2_. The stimulating effect of O_2_ in the propane pathway was shown to be largely due to increased amounts of free butyrate precursor released from FASII. O_2_ also has an indispensable role in the final reaction, as it serves as a co-substrate for the ADO-catalysed conversion of aldehydes to alkanes^7^. Excessive oxygen, on the other hand, was found to be detrimental for propane production. In part, this may be explained by the fact that hydrogen peroxide, a reactive by-product of oxygen, can inhibit ADO^23^. The overexpression of *E. coli* KatE, however, did not improve propane production. Another possibility is that ADO may be susceptible to other physiologically relevant radical oxidizing species, or the integrity of the Fe-S cluster in ferredoxin may be negatively influenced by elevated O_2_ concentrations. The latter would be surprising given that PetF originates from the oxygenic cyanobacteria *Synechocystis sp*. PCC 6803.

The experimental setup adopted for this study was chosen to demonstrate the feasibility of generating propane and to rapidly evaluate the influence of a series of physical and biochemical factors on propane synthesis. The downside to using sealed cultivation vessels was the inability to maintain stable O_2_ levels, and as demonstrated by the overall production profile of the optimized strain, poor yields are obtained as the majority of the consumed glucose ends up as biomass or alternate metabolites. With a controlled environment under steady-state conditions, we expect greater titres and continuous stable production to be achievable. For commercial production in an eventual future implementation, technical challenges in the coupling of a bioreactor with gas separation/collection technologies will also need to be addressed. Several technologies already exist for the separation of gases, such as cryogenic distillation^41^.

The fact that the propane-generating pathway reported in this manuscript is O_2_ tolerant, and driven principally by NADPH, ATP and reduced ferredoxin, will facilitate its transfer to photosynthetic microorganisms, which naturally accumulate these co-substrates. Recently, the expression of *E. coli* TesA and deletion of acyl-ACP synthase (slr1609) was shown to result in substantial accumulation of C12–C16 fatty acids^42^. As cyanobacteria effectively couple photosynthesis to the reduction of PetF, and already in most cases also harbour ADO^6^, only Tes4, CAR and Sfp will need to be introduced in order to enable propane biosynthesis, at least in theory. An alternative is to utilize the butyraldehyde-dependent butanol pathways that have recently been implemented in *Synechococcus elongatus* PCC 7942 (ref. 15). In fact, the *S. elongatus* PCC 7942 strains that produce butanol^15^,^43^ may already inadvertently produce propane, only that these previous studies did not collect and monitor for it.

In conclusion, an effective propane pathway would be useful not only for waste biomass recycling with fermentative biotechnology, but also in oxygenic photosynthetic biotechnological hosts, thereby allowing the direct conversion of solar into chemical energy. Although the development of a renewable propane pathway in this article only demonstrates a first concept for optimal matching of the chemical product with the entire process for production and utilization, we would argue that a similar approach will be useful when also other novel fuel targets are sought, in order to enhance chances for eventual adoption and large-scale commercialization.

## Methods

### Reagents and enzymes

Restriction enzymes were purchased from New England BioLabs. T4 ligase and DNA polymerase enzymes were purchased from Thermo-Scientific Fermentas. Oligonucleotides were from Eurofins MWG Operon. All chemicals for culture media were obtained from Sigma-Aldrich.

### Strains and DNA manipulation

*E. coli* DH5 alpha was used to propagate all plasmids routinely in lysogeny broth (LB) medium (10 g tryptone, 5 g yeast extract and 10 g NaCl per litre) at 37 °C, 150 r.p.m. shaking, overnight. *E. coli* BL21(DE3) was used for expression studies. The plasmids, genes and organisms used in this study are listed in Supplementary Table 3; the oligonucleotide primers for cloning and gene knockouts are listed in Supplementary Table 5.

### Gene knockouts

To construct the *yqhD* (GenBank ID: ACT44688.1) and *ahr* (GenBank ID: AAA97166.1) deletion mutants, an FLP recognition target flanked kanamycin resistance cassette was inserted using electroporation to the *E. coli* BL21 (DE3) host strain as described by Datsenko *et al.*^44^ The flanked resistance cassette and the target chromosomal gene were recombined using Red recombinase; the recombination was initiated by adding L-arabinose to the growth medium. The kanamycin selection markers were removed by Flp-mediated excision.

### Manipulation of plasmids using pET vectors

The pET-TPC plasmids were created by cloning synthetized genes encoding acyl-ACP-thioesterase from *Anaerococcus tetradius* (GenBank ID: EEI82564), *Bacteroides fragilis* (GenBank ID: CAH09236) and *Haemophilus influenzae* (GenBank ID: AAC22485.1) into a pET (Novagen, Amp^R^) backbone vector^10^ with genes coding for CAR from *Mycobacterium marinum* (GenBank ID: ACC40567.1) and its maturation factor phosphopantetheinyl transferase (Sfp) from *Bacillus subtilis* (GenBank ID: X65610.1) using *Nco*I and *Hin*dIII recognition sites.

### Manipulation of plasmids using pCDF vectors

*ADO* gene from *Prochlorococcus marinus* (GenBank ID: BX548175) was kindly provided by E. Neil G. Marsh (Department of Biological Chemistry, University of Michigan, USA) and subcloned into a pCDF-Duet vector (Novagen, Strep^R^) using *Nco*I and *Eco*RI restriction sites. The pCDF-PPC vector was created by cloning *petF*, *sfp* and CAR into a pCDF-Duet vector (Novagen, Strep^R^). The pCDF-FD3 plasmid was created by inserting the ADO to a pCDF-PetF vector using *Bam*HI and *Avr*II restriction sites; the restriction sites were introduced by PCR using the oligonucleotide primers listed in Supplementary Table 5.

### Manipulation of plasmids using pACYC vectors

Ferredoxin I gene (*petF*) from *Synechocystis sp.* PCC 6803 (GenBank ID: AAB72025.1) was synthetized by GenScript (USA) and subcloned into a pACYC vector (Novagen, Cam^R^). Genes encoding for catalase *katE* (GenBank ID: AAT48137.1) and NADPH:Ferredoxin/Flavodoxin-oxidoreductase *fpr* (GenBank ID: AAB03056.1) were subcloned from *E. coli* K12 (GenBank ID: AP009048) genome into a pACYC vector (Novagen, Cam^R^) using *Avr*II and *Hin*dIII/*Avr*II restriction sites, respectively.

### Media and cultivation for alkane production

The culture conditions were optimized for the parameters listed in Supplementary Table 4. Five millilitres of LB liquid media were inoculated from glycerol stocks (−80 °C) and incubated O/N at 37 °C (150 r.p.m.) in 12 ml Greiner polypropylene microbial culture tubes under aerobic conditions, with vent stoppers. Forty-five millilitres of M9 minimal (240.8 mg MgSO_4_, 11.1 mg CaCl_2_, 1 mg thiamine, 6.78 g Na_2_HPO_4_, 3 g NaH_2_PO_4_, 0.5 g NaCl, 1 g NH_4_Cl and 20 g glucose per litre) or TB media (12 g tryptone, 24 g yeast extract, 4 ml glycerol, 12.5 g K_2_HPO_4_, 2.3 g KH_2_PO_4_, and 20 g glucose per litre) were inoculated with a 10% (v/v; 5 ml) inoculum ratio in 250-ml Duran-Schott, baffled Erlenmeyer flasks closed with GL-45 oxygen permeable screw caps. The cultures were allowed to grow (37 °C, 200 r.p.m.) until OD_600_ was between 0.5 and 0.9, followed by addition of isopropyl β-D-1-thiogalactopyranoside to the final concentration of 0.5 mM (or as otherwise mentioned). The induced cultures were grown (30 °C, 200 r.p.m.) for 4 h (or as otherwise mentioned). The cells were pelleted (21 °C, 5,000 g, 10 min) and re-suspended either with 12.5 ml M9 or with TB media; 0.5 ml re-suspended culture were transferred to 2 ml gas-tight Agilent GC vials (Agilent) and incubated at room temperature typically for 3 h. Four parallel individual reactions were always prepared for each data point from the same main culture, and these were used to measure s.d. as shown in the result bar charts. In addition, consecutive experiments were designed to overlap, so that individual data sets and observed trends could also be compared between different reaction series. Additional oxygen (99.5%; AGA Gas) was supplemented into the culture headspace using a gas-tight syringe and a 0.3-mm injection needle (with a second needle as an outlet) through the GC-vial septum. The scaled-up experiments were done in an identical matter, but in 160 ml gas-tight serum bottles with 20 ml culture. The same protocol was used for heptane production. After the incubation period was over, the headspace of the vials was analysed for alkanes with GC–MS.

The initial evaluation of strains Tes4 and Tes4Car, for the production of acids and alcohols, was carried out in 250 ml shake-flasks with ample aeration, whereas all other subsequent reactions that produce gaseous alkanes were carried out in 2 ml gas chromatography vials sealed with gas-tight polytetrafluoroethylene (PTFE) septa.

### Alkane measurement

Alkane analysis was carried out with an Agilent 7890C GC with autosampler and 5975C inert MS (Agilent), and a Supelco Equity-1 (300 °C, 30 m × 0.32 mm × 1 μm) column. Ten microlitres of the headspace of 2 ml gas-tight GC vials were injected in 10:1 split mode (1.9 ml min^−1^ He flow, injector at 300 °C, 35–63 °C with 5 °C min^−1^). Propane eluted at ~1.2 min and was confirmed by comparison with a commercial standard (AGA Gas). Quantification was done by relating the GC–MS peak area (ion *m*/*z*=43) of the sample to the peak area of a 1% (v/v) commercial propane gas standard in N_2_, both of which were injected from the headspace of sealed culture vials. In all cases, four biological replicates were analysed in parallel to calculate the s.d. presented in the bar charts.

Heptane was analysed with the same GC–MS system as for propane, equipped with an Agilent Innowax 19091N-213 (260 °C, 30 m × 0.32 mm × 0.5 μm) column (Agilent). Ten microlitres of the headspace of 2 ml gas-tight GC vials were injected in 10:1 split mode (1.5 ml min^−1^ He flow, injector at 250 °C, 35 °C for 2 min then to 50 °C with 5 °C min^−1^). Heptane eluted at ~1.5 min and was confirmed with commercial standard (Sigma-Aldrich). The total amount of heptane in the reaction vessel (gas+liquid) was estimated by relating the GC–MS peak area (ion *m*/*z*=57) from the headspace sample of each reaction vessel with the peak areas of a series of dilutions prepared as follows: A known volume of commercial heptane in the liquid state was diluted in acetone to a concentration of 1 g of heptane per litre of acetone. A series of different volumes of the freshly prepared acetone/heptane mixture were thereafter added to 0.5 ml of TB media in 2 ml GC vials and the vessel was immediately closed. The heptane standards were incubated for 30 min with mixing followed by GC–MS analysis of the headspace and quantification.

### Alcohol and fatty acid measurement

The GC–MS system used for butanol and butyrate analysis was the same as described for heptane above. The supernatant of culture media was mixed with acetone (1:1) and injected to GC–MS (1.4 ml min^−1^ He flow, injector at 250 °C, 40–250 °C, 20 °C min^−1^). Butanol typically eluted at 5.5 min and butyrate eluted at 9.3 min; both were confirmed with commercial standards (Sigma-Aldrich); quantification was done by comparing the peak areas (ion *m*/*z*=56 for butanol and 87 for butyrate) to those of freshly prepared standards in acetone.

### Growth rate measurement

The growth rate of the cultures was estimated by the optical density (600 nm) in 10 mm single-use cuvettes using a Thermo-Scientific Genesys 10S or Genesys 2 UV–vis spectrophotometer (Thermo-Scientific).

## Author contributions

P.K., A.P., M.K.A., K.T. and P.R.J. designed the experiments. P.K., A.P., M.K.A. and K.T. performed the experiments. P.K., A.P., M.K.A. and P.R.J. wrote the manuscript.

## Additional information

**How to cite this article:** Kallio, P. *et al.* An engineered pathway for the biosynthesis of renewable propane. *Nat. Commun.* 5:4731 doi: 10.1038/ncomms5731 (2014).

## Supplementary Material

Supplementary Information Supplementary Figures 1-20, Supplementary Tables 1-5 and Supplementary References
