# Supplementary material for: An engineered pathway for the biosynthesis of renewable propane
Source: Nat Commun. 2014 Sep 2;5:4731. doi: 10.1038/ncomms5731 (PMC4164768; doi:10.1038/ncomms5731)
Supplement: Supplementary Information — Supplementary Figures 1-20, Supplementary Tables 1-5 and Supplementary References [file ncomms5731-s1.pdf]

SUPPLEMENTARY INFORMATION

SUPPLEMENTARY FIGURES

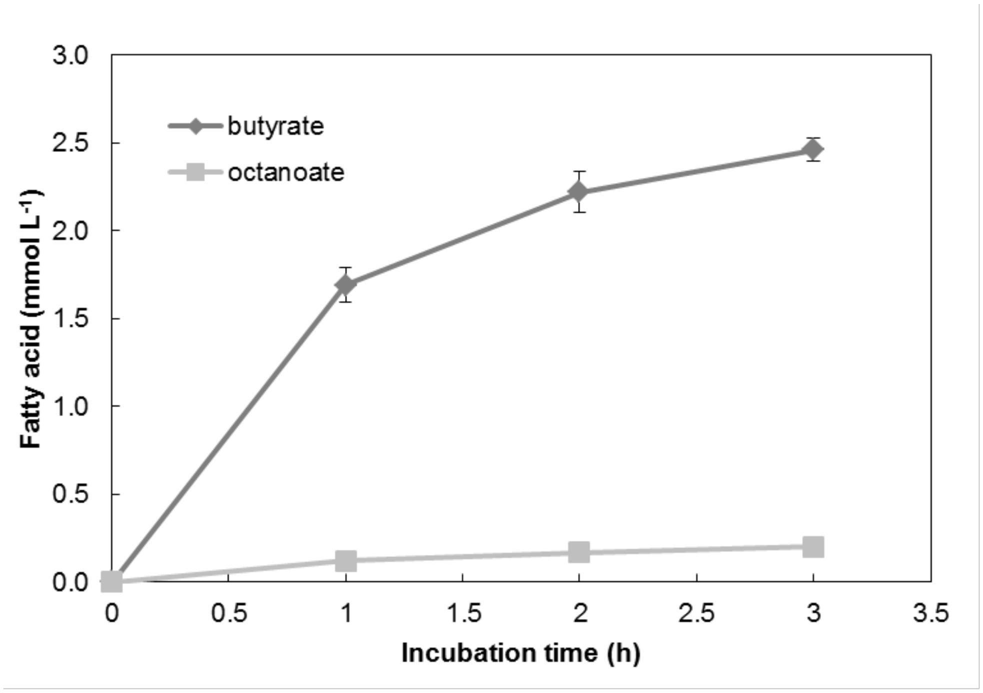

Supplementary Figure 1: Fatty acid production time-course analysis of Tes4 strain. Fatty acid production was evaluated at 80% v/v oxygen concentration with Tes4 (BL21 (DE3) pET-T4) in 2 ml gas-tight GC vials in an identical matter as propane production experiments were carried out. Samples were collected; fatty acids were extracted from the supernatant and analyzed by GC-MS using the method described in the Materials and Methods. Error bars represent standard deviation (n = 4); details of the plasmids are described in Supplementary Table 3.

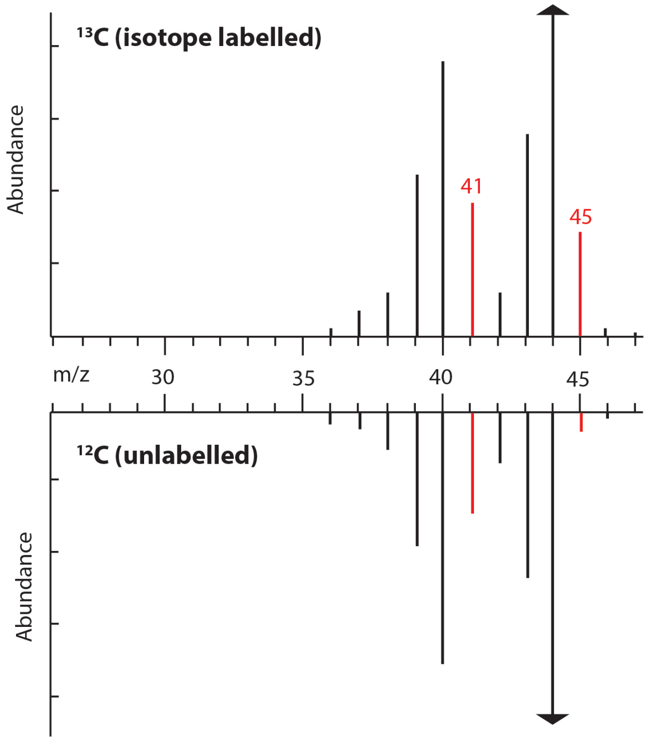

Supplementary Figure 2: Biologically produced propane and incorporation of ^{13}C label. Mass spectra from cultivation using 20g/L 40% ^{13}C labeled glucose versus normal glucose. The +1 m/z fragments 41 and 45 which are more abundant in the ^{13}C sample are highlighted in red.

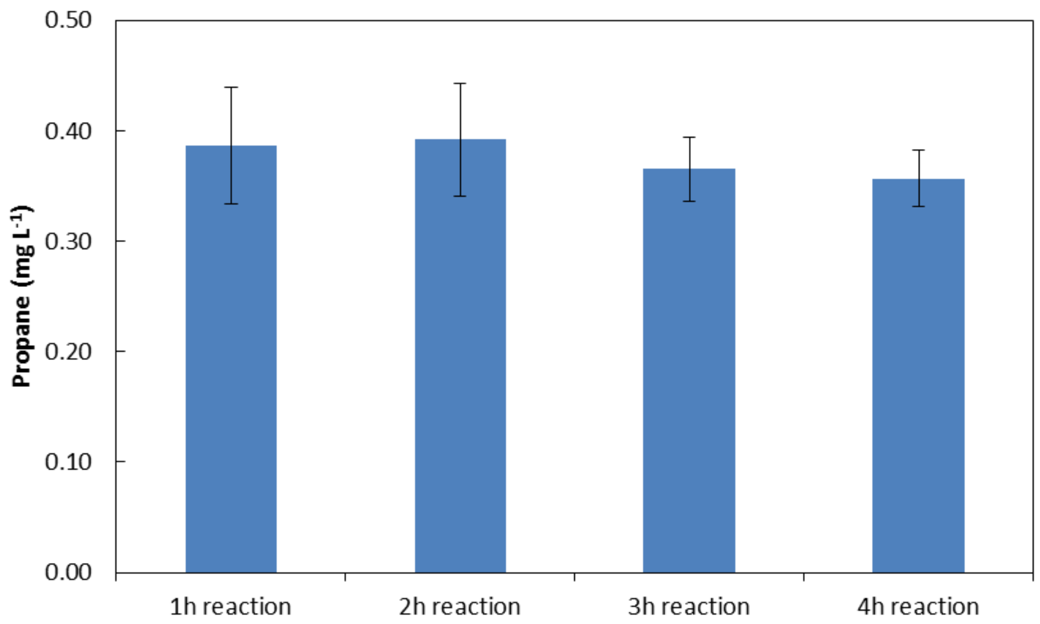

Supplementary Figure 3: Propane production time-course analysis of Pro strain. Propane production was done at atmospheric levels of oxygen with Pro (BL21 (DE3) pET-TPC4; pCDF-ADO; pACYC-petF). Error bars represent standard deviation (n = 4); details of the plasmids are described in Supplementary Table 3.

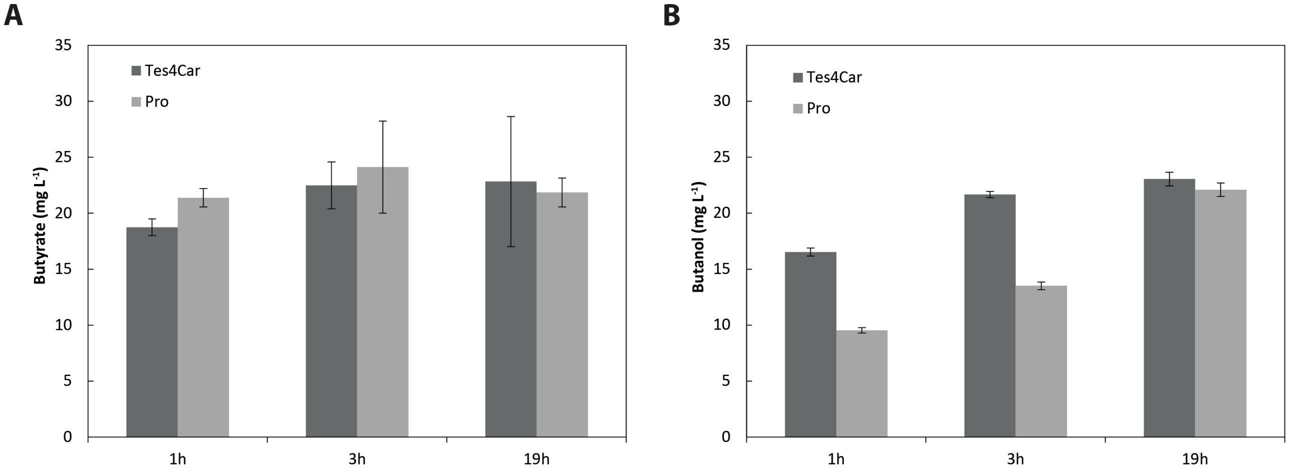

Supplementary Figure 4: Metabolic by-product production time-course analysis of Pro and Tes4Car strains. (**A**) Butyrate and (**B**) butanol production under atmospheric oxygen levels was evaluated, using Pro strain (BL21 (DE3) pET-TPC4; pCDF-ADO; pACYC-petF) and Tes4Car (BL21 (DE3) pET-TPC4). Strain Tes4Car was lacking ADO and petF enzymes. Error bars represent standard deviation (n = 4); details of the plasmids are described in Supplementary Table 3.

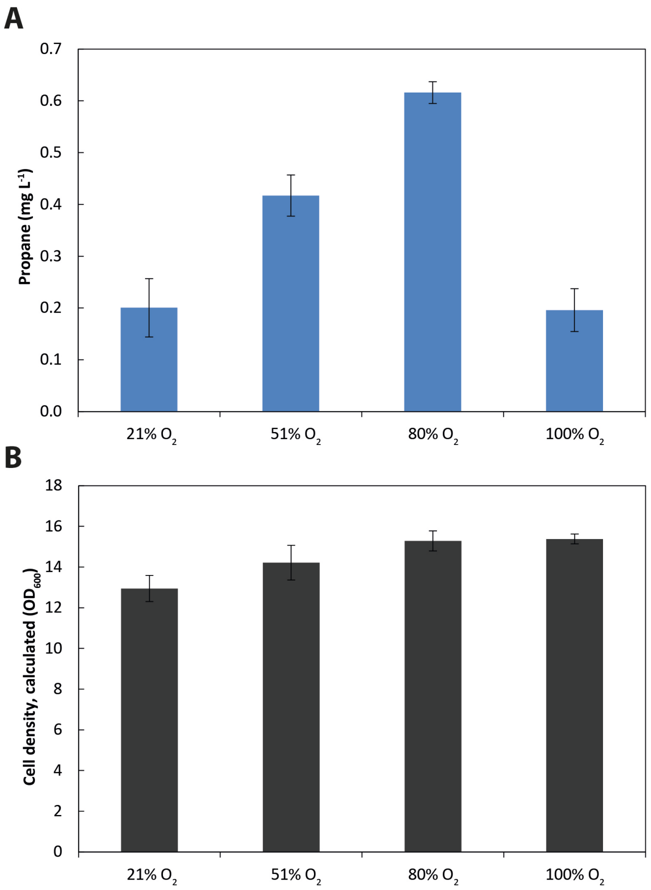

Supplementary Figure 5: The effect of increased oxygen concentration on propane production and cell density of Pro strain. (**A**) Propane production was evaluated in 2 ml gas-tight vials using Pro strain (BL21 (DE3) pET-TPC4; pCDF-ADO; pACYC-petF) and different oxygen concentration (21, 51, 80 and 100% v/v). (**B**) Cell density was measured at 600 nm from the same samples (the represented values are multiplied with the dilution rate). Error bars represent standard deviation (n = 4); details of the plasmids are described in Supplementary Table 3.

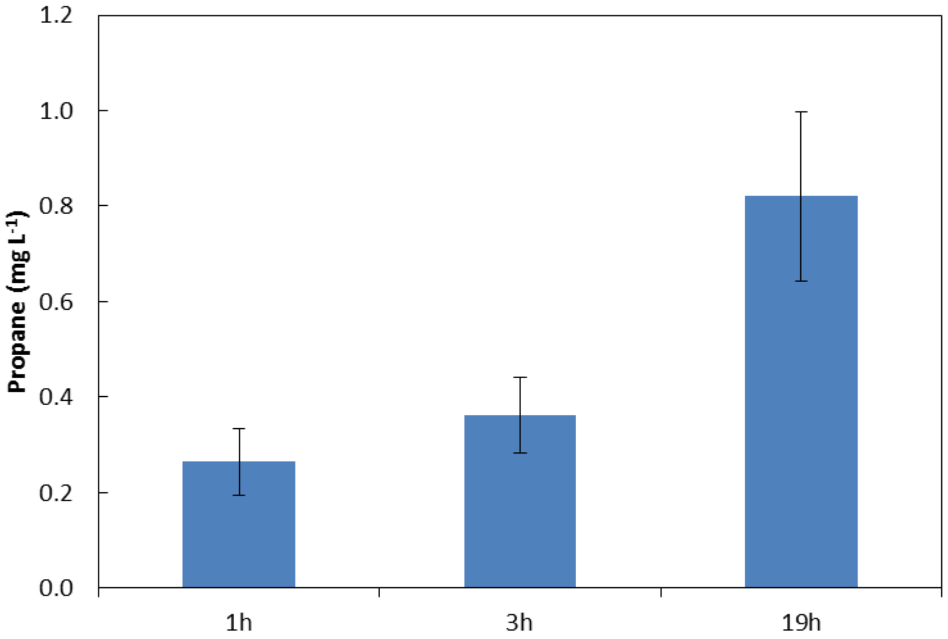

Supplementary Figure 6: The effect of increased reaction headspace on propane production time of Pro strain. Propane production was evaluated in 160 ml serum bottles instead of 2 ml vial with an increased incubation time in a time course analysis using Pro strain (BL21 (DE3) pET-TPC4; pCDF-ADO; pACYC-petF). Error bars represent standard deviation (n = 4); details of the plasmids are described in Supplementary Table 3.

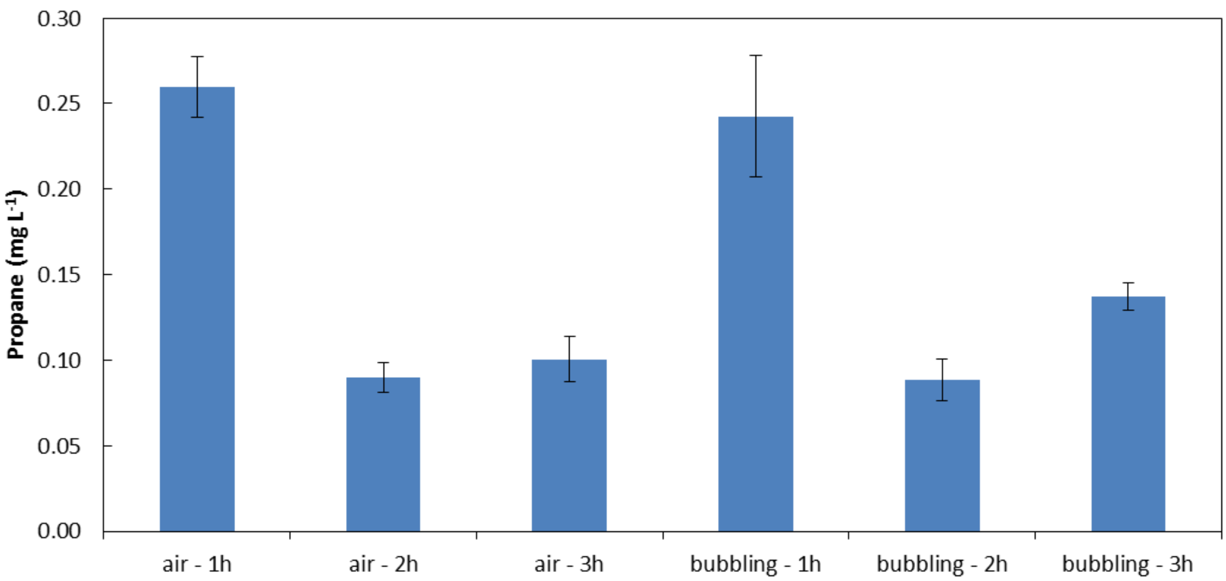

Supplementary Figure 7: Effect of reaction vial headspace regeneration on propane production of Pro strain. Vials opened and resealed at one-hour intervals, with and without blowing the culture with air, followed by 1 h incubation and propane quantitation using Pro strain (BL21 (DE3) pET-TPC4; pCDF-ADO; pACYC-petF). Error bars represent standard deviation (n = 4); details of the plasmids are described in Supplementary Table 3.

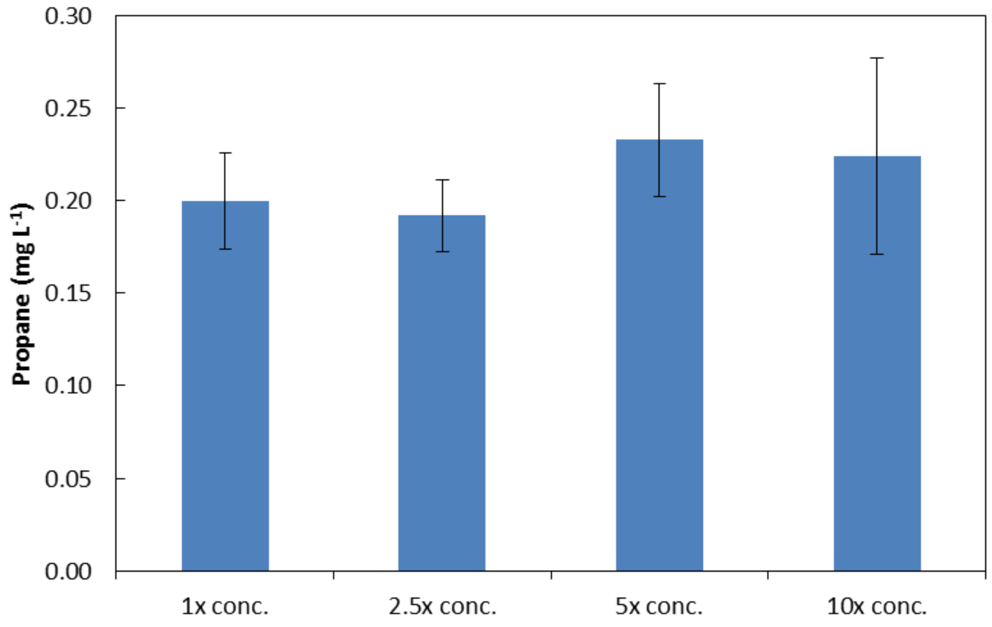

Supplementary Figure 8: The effect of increased cell concentration on propane production of Pro strain. Propane production was evaluated in 2 ml gas-tight vials using Pro strain (BL21 (DE3) pET-TPC4; pCDF-ADO; pACYC-petF) and different cell concentration (1, 2.5, 5 and 10 times concentrated). Cells were centrifuged and re-suspended with production media, prior to sealing in gas-tight vials. Error bars represent standard deviation (n = 4); details of the plasmids are described in Supplementary Table 3.

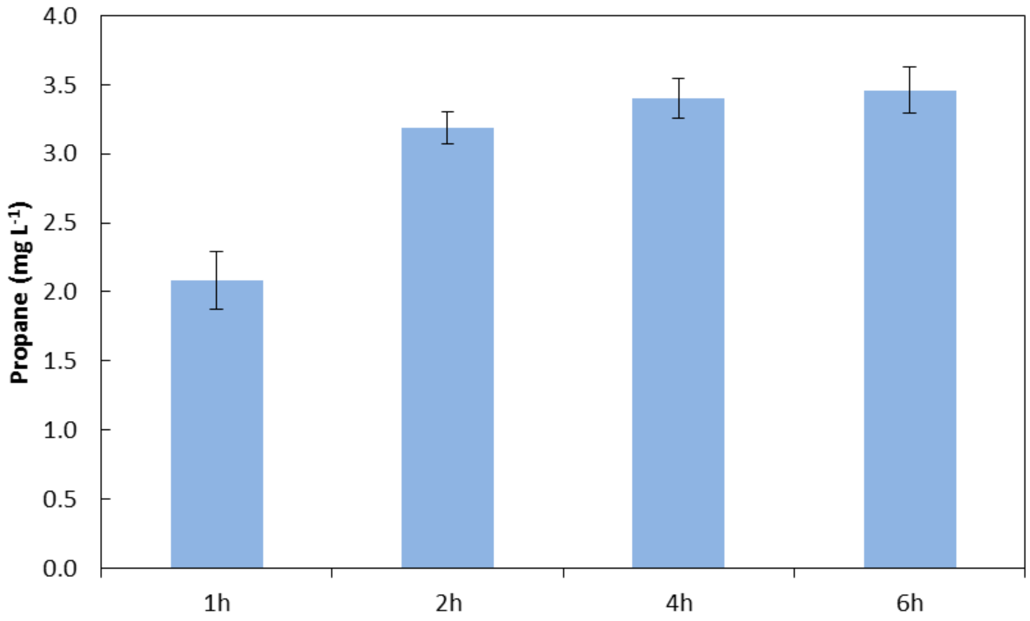

Supplementary Figure 9: Propane production time-course analysis of CarAdo strain. Propane was produced by feeding butyrate under atmospheric oxygen levels, using CarAdo strain (BL21 (DE3) pET28-ADO; pCDF-PPC). Error bars represent standard deviation (n = 4); details of the plasmids are described in Supplementary Table 3.

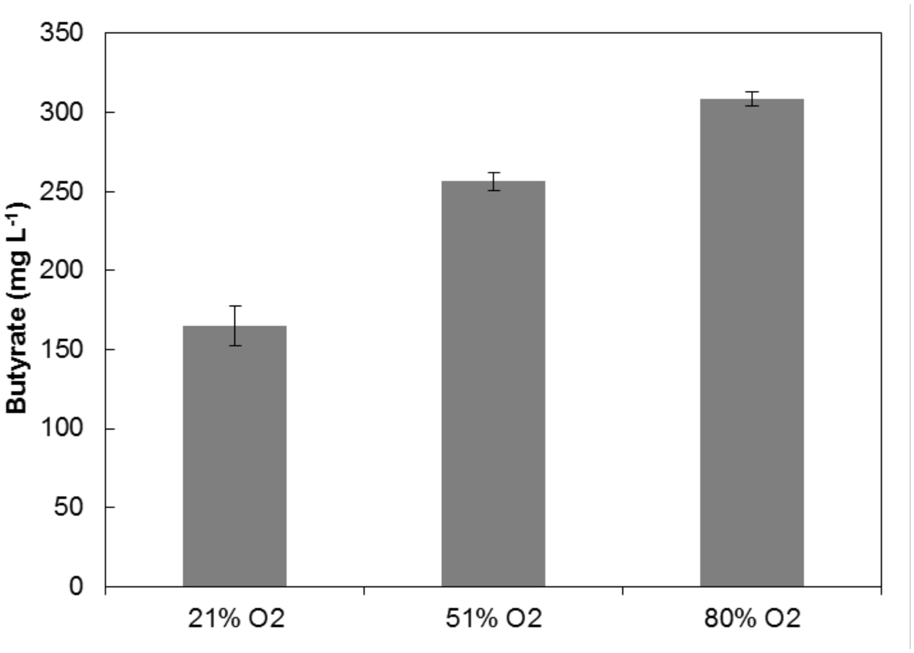

Supplementary Figure 10: The effect of oxygen concentration on butyric acid production of Tes4 strain. Butyrate production was evaluated at 21, 51 and 80% v/v oxygen concentrations with Tes4 (BL21 (DE3) pET-T4) in 2 ml gas-tight GC vials with 3h incubation time in an identical matter as propane production experiments were carried out. Samples were collected; butyrate was extracted from the supernatant and analyzed by GC-MS using the method described in the Materials and Methods. Error bars represent standard deviation (n = 4); details of the plasmids are described in Supplementary Table 3.

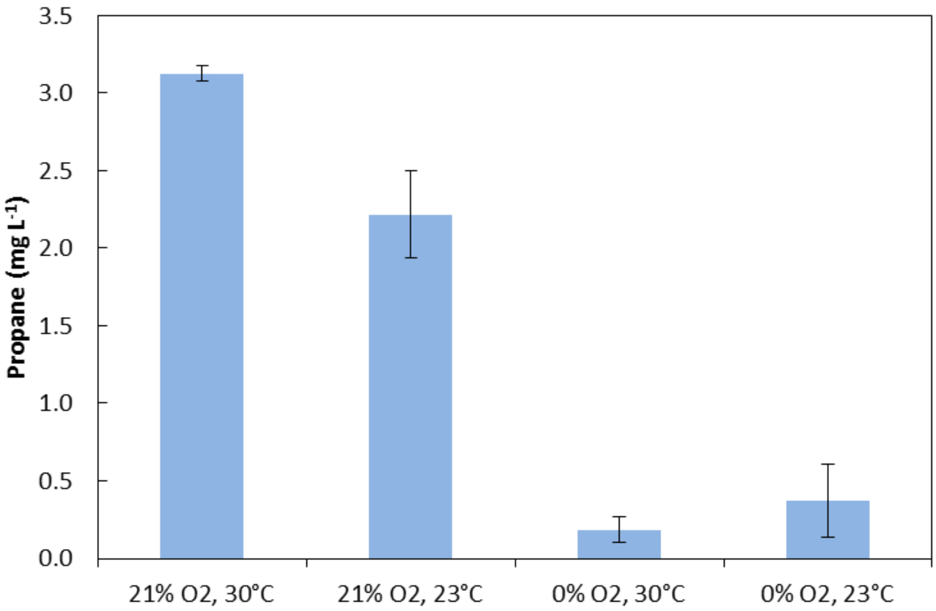

Supplementary Figure 11: Propane production of CarAdo strain fed with butyrate under aerobic and anaerobic conditions. Propane production was evaluated at 23 and 30°C under normal and nitrogen atmosphere (0% O_2), using CarAdo strain (BL21 (DE3) pET28-ADO; pCDF-PPC). Error bars represent standard deviation (n = 4); details of the plasmids are described in Supplementary Table 3.

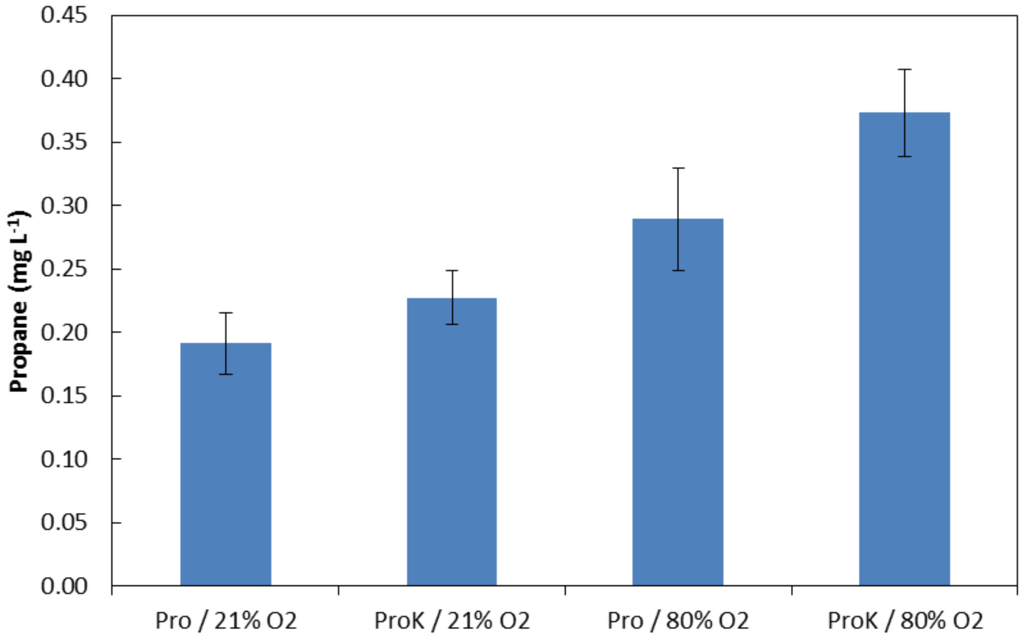

Supplementary Figure 12: The effect of KatE overexpression on propane production. Propane production was evaluated with Pro (BL21 (DE3) pET-TPC4; pCDF-ADO; pACYC-petF) and ProK (BL21 (DE3) pET-TPC4; pCDF-ADO; pACYC-petF-katE) strains under 21% v/v and 80% v/v oxygen concentrations. Error bars represent standard deviation (n = 4); details of the plasmids are described in Supplementary Table 3.

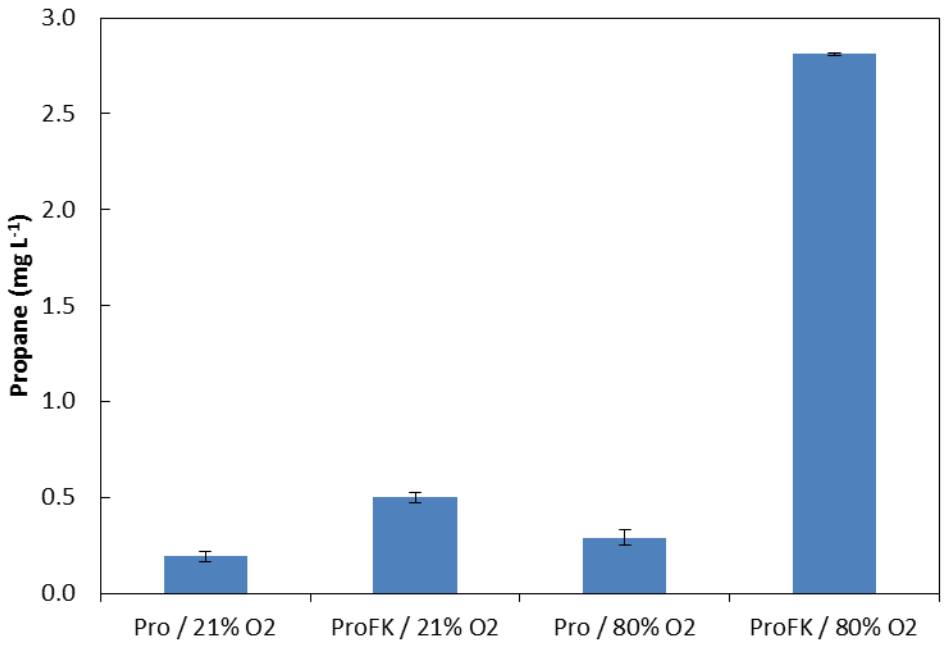

Supplementary Figure 13: The effect of fpr and KatE overexpression on propane production. Propane production was evaluated with Pro (BL21 (DE3) pET-TPC4; pCDF-ADO; pACYC-petF) and ProFK (BL21 (DE3) pET-TPC4; pCDF-ADO; pACYC-petF-fpr-katE) strains under 21% v/v and 80% v/v oxygen concentrations. Error bars represent standard deviation (n = 4); details of the plasmids are described in Supplementary Table 3.

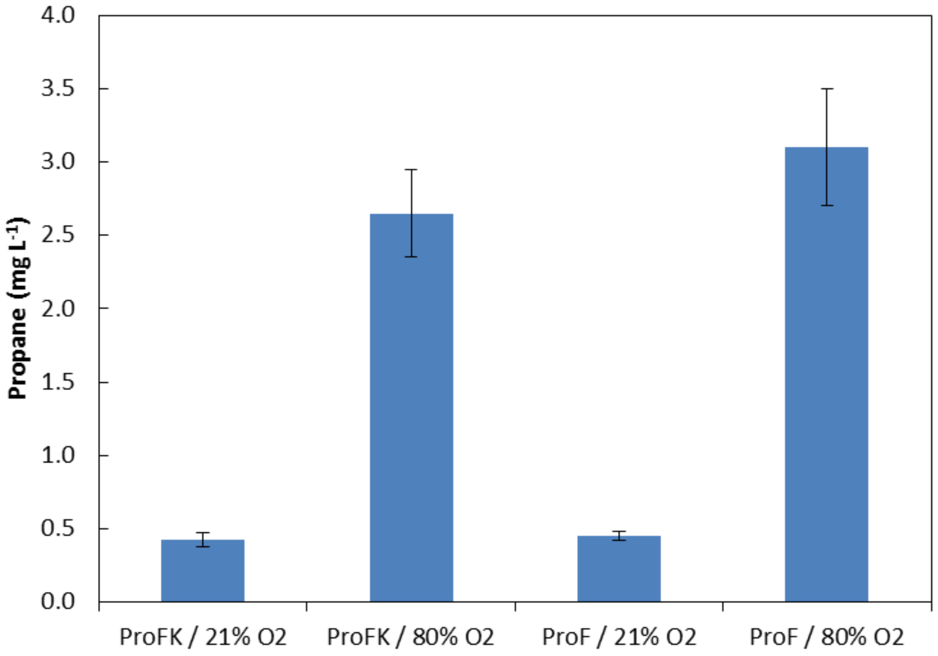

Supplementary Figure 14: The effect of KatE overexpression on propane production. Propane production was evaluated with ProF (BL21 (DE3) pET-TPC4; pCDF-ADO; pACYC-petF-fpr) and ProFK (BL21 (DE3) pET-TPC4; pCDF-ADO; pACYC-petF-fpr-katE) strains under 21% v/v and 80% v/v oxygen concentrations. Error bars represent standard deviation (n = 4); details of the plasmids are described in Supplementary Table 3.

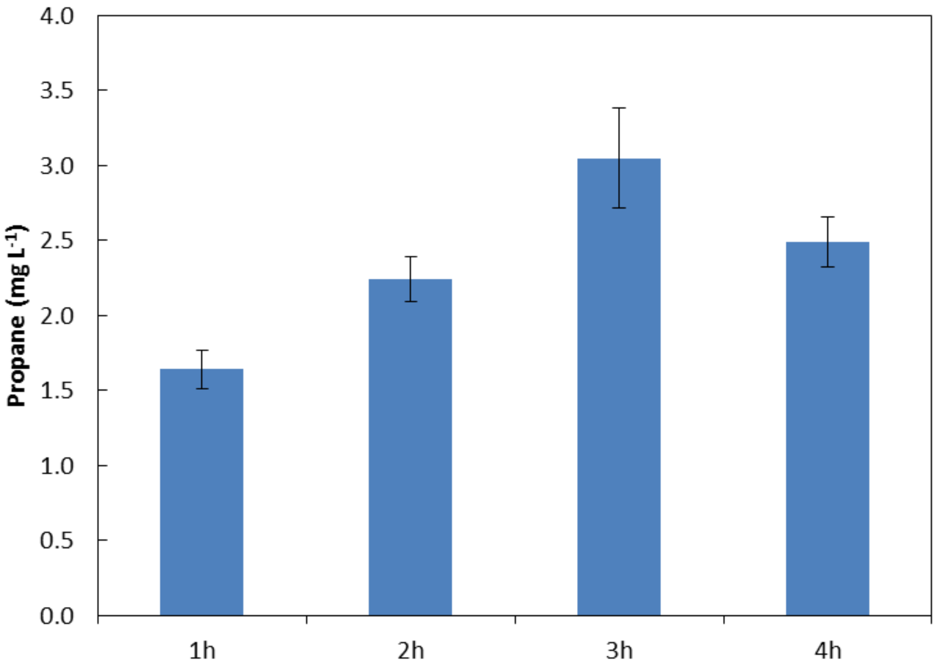

Supplementary Figure 15: Propane production time-course analysis of ProFK strain. Propane was produced by overexpressing Fpr and KatE under atmospheric oxygen levels, using ProFK strain (BL21 (DE3) pET-TPC4; pCDF-ADO; pACYC-petF-fpr-katE). Error bars represent standard deviation (n = 4); details of the plasmids are described in Supplementary Table 3.

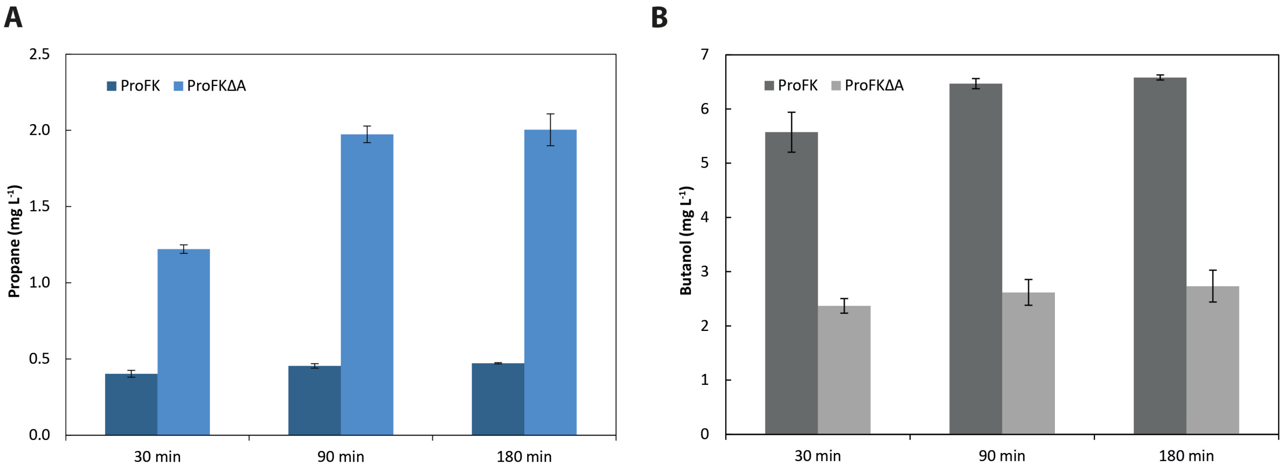

Supplementary Figure 16: Propane and butanol production of aldehyde reductase knock-out with fpr and katE overexpression. (**A**) Propane and (**B**) butanol production was evaluated with ProFK (BL21 (DE3) pET-TPC4; pCDF-ADO; pACYC-petF-fpr-katE) and ProFKΔA (BL21 (DE3) ΔyqhD Δahr pET-TPC4; pCDF-ADO; pACYC-petF-fpr-katE) strains under 21% v/v oxygen concentration at 30, 90 and 180 min time points. Error bars represent standard deviation (n = 4); details of the plasmids are described in Supplementary Table 3.

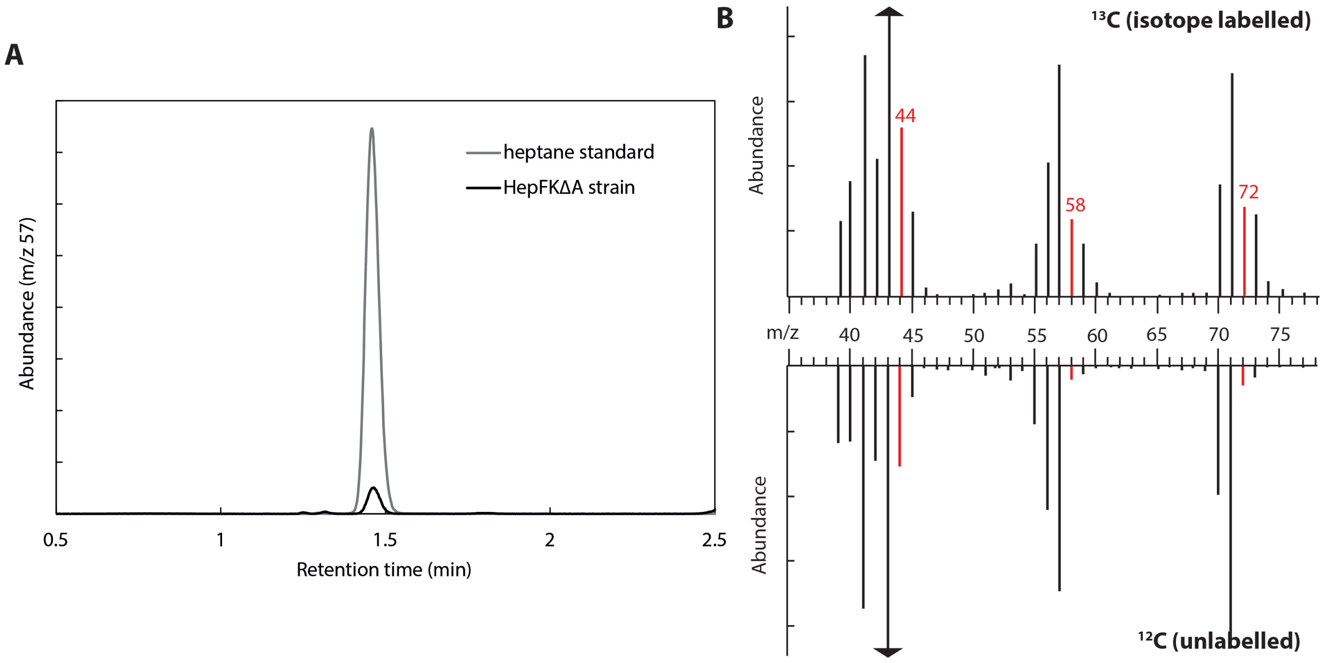

Supplementary Figure 17: Biologically produced heptane and incorporation of ^{13}C label. (**A**) Overlay GC-MS chromatogram of heptane standard and the product peak of HepFKΔA (BL21 (DE3) ΔyqhD Δahr pET-TPC3; pCDF-ADO; pACYC-petF-fpr-katE) strain. The mass spectra were filtered for m/z 57 using standard and HepFKΔA (Hep+fpr+katE ΔyqhD Δahr) strain. The parameters of the analysis are described in the Materials and Methods section. (**B**) Incorporation of ^{13}C label in heptane from glucose: Ion fragmentation pattern of heptane (M=100.2 g mol^{-1}) produced using TB medium with 20g/L 40% ^{13}C labeled glucose versus normal glucose. The +1 m/z fragments 44, 58 and 72 which are more abundant in the ^{13}C sample are highlighted in red.

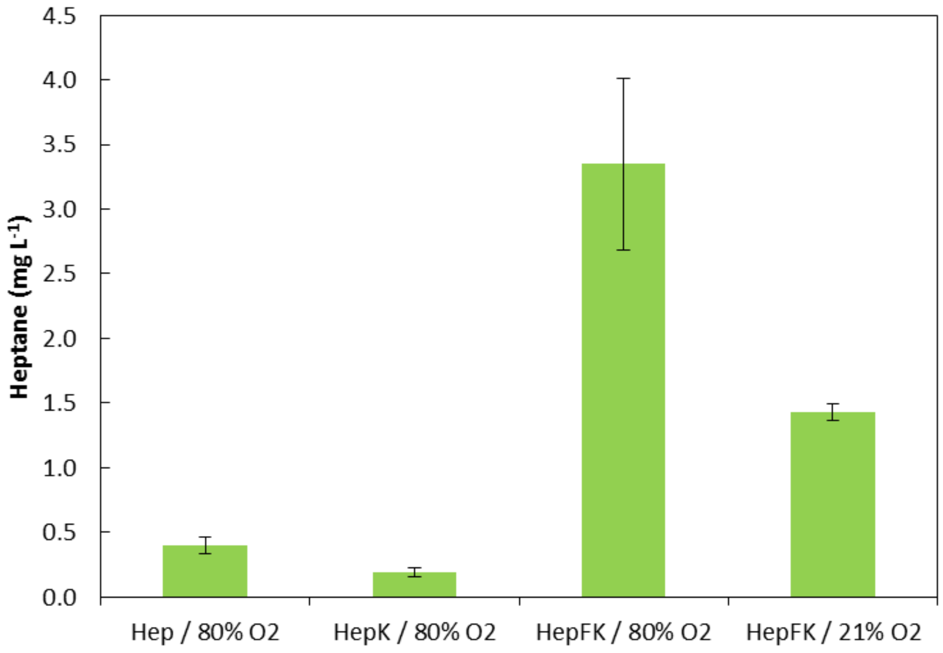

Supplementary Figure 18: The effect of fpr and KatE overexpression on heptane production. Heptane production was evaluated with Hep (BL21 (DE3) pET-TPC3; pCDF-ADO; pACYC-petF), HepK (BL21 (DE3) pET-TPC3; pCDF-ADO; pACYC-petF-katE) and HepFK (BL21 (DE3) pET-TPC3; pCDF-ADO; pACYC-petF-fpr-katE) strains under 21% v/v and 80% v/v oxygen concentrations. Error bars represent standard deviation (n = 4); details of the plasmids are described in Supplementary Table 3.

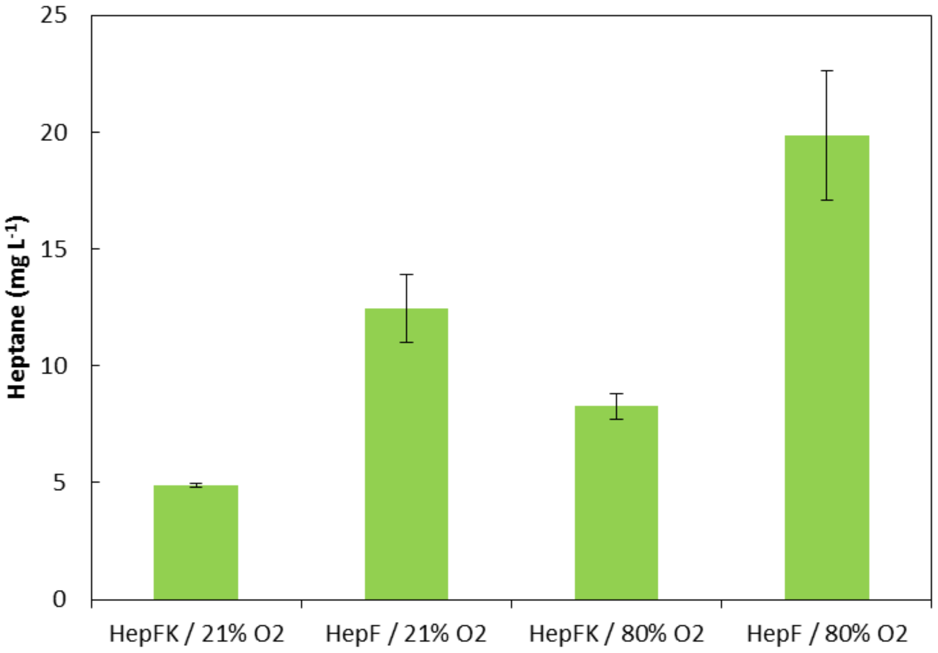

Supplementary Figure 19: The effect of KatE and fpr co-expression on heptane production. Heptane production was evaluated with HepF (BL21 (DE3) pET-TPC3; pCDF-ADO; pACYC-petF-fpr) and HepFK (BL21 (DE3) pET-TPC3; pCDF-ADO; pACYC-petF-fpr-katE) strains under 21% v/v and 80% v/v oxygen concentrations. Error bars represent standard deviation (n = 4); details of the plasmids are described in Supplementary Table 3.

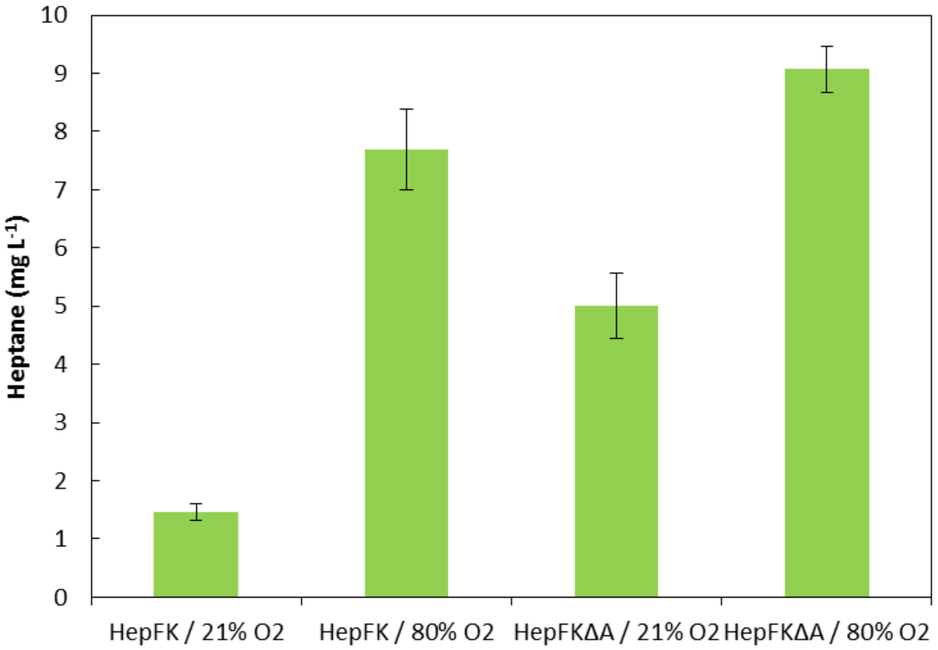

Supplementary Figure 20: The effect of aldehyde reductase knockout on heptane production. Heptane production was evaluated with HepFK (BL21 (DE3) pET-TPC3; pCDF-ADO; pACYC-petF-fpr-katE) and HepFKΔA (BL21 (DE3) ΔyqhD Δahr pET-TPC3; pCDF-ADO; pACYC-petF-fpr-katE) strains under 21 and 80% v/v oxygen concentrations. Error bars represent standard deviation (n = 4); details of the plasmids are described in Supplementary Table 3.

SUPPLEMENTARY TABLES

Supplementary Table 1: Biologically produced hydrocarbons and properties of gaseous fuels

Biologically relevant alkanes/alkenes	Physical state at STP	Boiling point	References
Methane	Gas	-161°C	1
Ethane	Gas	-89°C	2
Propane	Gas	-42°C	**This study**
Pentane	Liquid	36°C	3
Heptane	Liquid	98°C	**This study**
Octane	Liquid	121°C	4
Nonane	Liquid	151°C	4
Undecane	Liquid	196°C	5
Dodecane	Liquid	216°C	4
Tridecane	Liquid	235°C	4, 5, 6, 7
Tetradecane	Liquid	254°C	4
Pentadecane	Liquid	271°C	6
Pentadecene	Liquid	268°C	5, 6, 7
Hexadecane	Liquid	287°C	6
Hexadecene	Liquid	285°C	6
Heptadecane	Solid	316°C	6
Heptadecene	Liquid	300°C	5, 6, 7

**Fuel**	**Ideal work of liquefaction** (kJ/kg)	**Higher heating value** (kJ/kg)	**Work to liquefy per available energy** % (J/J)
H_2	12019	141800	8.48
CH_4	1091	55530	1.97
C_3H_8	140.4	50322	0.28

The ideal work of liquefaction, values obtained from Barron et al.[8] (Table 3.1, p. 63), is a theoretical estimate of the amount of work that is needed to liquefy the gas. The higher heating value is an estimate of the total energy released upon complete combustion[9]. The theoretical work required to liquefy the gas is compared to the total energy available upon combustion.

Supplementary Table 2: List of bacterial strains. The abbreviations containing Pro and Hep refer to strains producing propane, heptane respectively; Tes4 is producing butyrate; TesCar is producing butanol; CarAdo is referred to the strain used in the butyrate feeding study for propane production.

**Strain**	**Host**	**Plasmids**
Tes3	*E. coli* BL21 (DE3)	pET-T3
Tes4	*E. coli* BL21 (DE3)	pET-T4
Tes4Car	*E. coli* BL21 (DE3)	pET-TPC4
Pro	*E. coli* BL21 (DE3)	pET-TPC4; pCDF-ADO; pACYC-petF
ProF	*E. coli* BL21 (DE3)	pET-TPC4; pCDF-ADO; pACYC-petF-fpr
ProK	*E. coli* BL21 (DE3)	pET-TPC4; pCDF-ADO; pACYC-petF-katE
ProFK	*E. coli* BL21 (DE3)	pET-TPC4; pCDF-ADO; pACYC-petF-fpr-katE
ProFΔA	*E. coli* BL21 (DE3) ΔyqhD Δahr	pET-TPC4; pCDF-ADO; pACYC-petF-fpr
ProKΔA	*E. coli* BL21 (DE3) ΔyqhD Δahr	pET-TPC4; pCDF-ADO; pACYC-petF-katE
ProFKΔA	*E. coli* BL21 (DE3) ΔyqhD Δahr	pET-TPC4; pCDF-ADO; pACYC-petF-fpr-katE
CarAdo	*E. coli* BL21 (DE3)	pET28-ADO; pCDF-PPC
Hep	*E. coli* BL21 (DE3)	pET-TPC3; pCDF-ADO; pACYC-petF
HepF	*E. coli* BL21 (DE3)	pET-TPC3; pCDF-ADO; pACYC-petF-fpr
HepK	*E. coli* BL21 (DE3)	pET-TPC3; pCDF-ADO; pACYC-petF-katE
HepFK	*E. coli* BL21 (DE3)	pET-TPC3; pCDF-ADO; pACYC-petF-fpr-katE
HepFΔA	*E. coli* BL21 (DE3) ΔyqhD Δahr	pET-TPC3; pCDF-ADO; pACYC-petF-fpr
HepKΔA	*E. coli* BL21 (DE3) ΔyqhD Δahr	pET-TPC3; pCDF-ADO; pACYC-petF-katE
HepFKΔA	*E. coli* BL21 (DE3) ΔyqhD Δahr	pET-TPC3; pCDF-ADO; pACYC-petF-fpr-katE

Supplementary Table 3: Detailed plasmid list used for the experiments

Plasmid name	Protein	Organism	Protein	Organism	Protein	Organism
pET-T3	TE3	Anaerococcus tetradius				
pET-T4	TE4	Bacteroides fragilis				
pET-TPC3	TE3	Anaerococcus tetradius	Sfp	Bacillus subtilis (strain 168)	CAR	Mycobacterium marinum
pET-TPC4	TE4	Bacteroides fragilis	Sfp	Bacillus subtilis (strain 168)	CAR	Mycobacterium marinum
pET28-ADO	ADO	Prochlorococcus marinus				
pCDF-ADO	ADO	Prochlorococcus marinus				
pCDF-PPC	PetF	Synechocystis sp. PCC 6803	Sfp	Bacillus subtilis (strain 168)	CAR	Mycobacterium marinum
pACYC-PetF	PetF	Synechocystis sp. PCC 6803				
pACYC-PetF-KatE	PetF	Synechocystis sp. PCC 6803	KatE	Escherichia coli		
pACYC-PetF-Fpr-KatE	PetF	Synechocystis sp. PCC 6803	Fpr	Escherichia coli	KatE	Escherichia coli

Supplementary Table 4: Optimization of the culture conditions. Various culture conditions and expression parameters were optimized for propane production using the strain Pro (BL21 (DE3) pET-TPC4; pCDF-ADO; pACYC-petF). The selected default conditions (right column) were used in the assays for analyzing the performance of the propane and heptane pathways in this study. The inducer concentration and cell density (*) varied between some experimental setups.

**Conditions**	**Tested parameters**	**Optimal/Default**
Cell density at induction (OD_{600nm})	0.25, 0.5, 1, 2	0.5-0.9 *
IPTG concentration	0.05mM, 0.25mM, 0.5mM, 1mM	0.25-1 *
Induction temperature	21°C, 30°C, 37°C	30°C
Induction time	2h, 4h, 6h	4h
Glucose concentration	5g/L, 10g/L, 25g/L	20g/L
Reaction culture volume in 2.1ml vial	0.25ml, 0.5ml, 1ml	0.5ml
Reaction cell density (concentration)	1x, 2x, 5x, 10x	4x
Reaction temperature	21°C, 30°C, 37°C	21°C
Reaction time	1h, 2h, 4h, 6h, 19h	3h

Supplementary Table 5: Oligonucleotide primers used in the experiments. Please refer to Materials and Methods for more details

Primer	Sequence
Tes3_FW	ACTTCACCATGGGCTAAGGTACCTAATTAATTAAAAAGGAG
Tes3_RE	ACCATCAAGCTTTTTAATTAATTACACGTTAGTTTTAATTTTCCCCAAAC
Tes4_RE	ACCATCAAGCTTTTTAATTAATTAAACAAATTTCACTTTGGCGC
Yqhd_del_FW	GCAGATCGTTCTCTGCCCTCATATTGGCCCAGCAAAGGGAGCAAGTAATGATTCCGGGGATCCGTCGACC
Yqhd_del_RE	CGAAAACGAAAGTTTGAGGCGTAAAAAGCTTAGCGGGCGGCTTCGTATATTGTAGGCTGGAGCTGCTTCG
Ahr_del_FW	GCCCTGCCATGCTCTACACTTCCCAAACAACACCAGAGAAGGACCAAAAAGTGTAGGCTGGAGCTGCTTCG
Ahr_del_RE	GAATATGTGCGAAAGAGGGCAGCGCCTCAGATCAGCGCTGCGAATGATTTATTCCGGGGATCCGTCGACCTG
KatE_FW	ATATCCTAGGTCATGAGGAGGTTTGGAATGTCGCAACATAACGAAAAGAACCCAC
KatE_RE	ATATCCTAGGTCAGGCAGGAATTTTGTCAATCTTAGGAA
Fpr_FW	AATATTAAGCTTTAAATGATTGAAGGAGGAAAAAATGGCTGATTGGGTAACAGGCAAAGTC
Fpr_RE	AATATTCCTAGGTTACCAGTAATGCTCCGCTGTCATATGG
ADO_BamHI_FW	ATATGGATCCGCGCGGCAGCCA
ADO_AvrII_RE	ATATCCTAGGTTAAGAAACCAGGGCCGCTGCG

SUPPLEMENTARY REFERENCES

1. Thauer, R. K. Biochemistry of methanogenesis: a tribute to Marjory Stephenson. 1998 Marjory Stephenson Prize Lecture. *Microbiology* **144,** 2377-2406.
2. Belay, N. & Daniels, L. Production of ethane, ethylene, and acetylene from halogenated hydrocarbons by methanogenic bacteria. *Appl. Environ. Microbiol.* **53,** 1604-1610 (1987).
3. Blazeck, J., L. Liu, Knight, R. & Alper, H. S. Heterologous production of pentane in the oleaginous yeast *Yarrowia lipolytica*. *J. Biotechnol*. **165,** 184-194 (2013).
4. Choi, Y. J. & S. Y. Lee. Microbial production of short-chain alkanes. *Nature* **502,** 571-574 (2013).
5. Akhtar, M. K., Turner, N. J. & Jones, P.R. Carboxylic acid reductase is a versatile enzyme for the conversion of fatty acids into fuels and chemical commodities. *Proc. Natl. Acad. Sci. U.S.A.* **110,** 87-92 (2013).
6. Howard, T. P. et al. Synthesis of customized petroleum-replica fuel molecules by targeted modification of free fatty acid pools in *Escherichia coli*. *Proc. Natl. Acad. Sci. U.S.A.* **110,** 7636-7641 (2013).
7. Schirmer, A., Rude, M. , Li, X., Popova, E. & del Cardayre, S. Microbial biosynthesis of alkanes. *Science* **329,** 559-562 (2010).
8. Barron, R.F. *Cryogenic Systems, 2nd ed* 53 (Oxford University Press, New York, 1985).
9. Burgess, D.R. Thermochemical data. In: Linstrom, P.J., Mallard, W.G. (eds.) *NIST Chemistry WebBook, NIST Standard Reference Database Number 69* (National Institute of Standards and Technology, Gaithersburg MD, 20899, http://webbook.nist.gov, 2012).
